# Colorimetric Determination of Glucose in Urine

**Published:** 1915-05

**Authors:** 


**Colorimetric Determination of Glucose in Urine.** A. F. Dimmock, Am. Med. The urine is diluted 20 times. A 1:3 solution of potassium carbonate is filtered and diluted so as to make 1:4. 1 part of the latter is added to 2 of the diluted urine boiled carefully for 3 minutes, avoiding boiling over, and diluted to 50 or 100 c.c. A half per cent. solution of pure glucose is treated in the same way and the tints are matched by adding water to the glucose solution. Knowing the dilution of the glucose solution and of the urine, the calculation is easily made. (Note: All such simple tests impress us as more troublesome and less reliable than the burette method. Get a Fehling or Benedict or other reagent from a regular chemist.

Such solutions are standardized to represent a definite quantity of dextrose per c.c. and the test is simple, the calculations easy and the result quite accurate. Why be afraid of the burette?. Ed.)

